# Toward Precision Vaccinology for Mpox: Rational Antigen Design, Next-Generation Platforms, and Immune Correlates of Protection

**DOI:** 10.3390/tropicalmed11090244

**Published:** 2026-08-26

**Authors:** Yithenthrathevinair K Paramasivam, Nur Syafiqah Mohamad Nasir, Mohd Zulkifli Salleh

**Affiliations:** Department of Medical Microbiology and Parasitology, School of Medical Sciences, Universiti Sains Malaysia Health Campus, Kota Bharu 16150, Malaysia; yithenthra10@gmail.com (Y.K.P.); nursyafiqahmohamadnasir@gmail.com (N.S.M.N.)

**Keywords:** mpox, precision vaccinology, antigen design, delivery platforms, correlates of protection

## Abstract

Mpox has emerged as a global public health concern, highlighting the limitations of traditional vaccinia-based vaccination strategies and the urgent need for precision vaccinology approaches. Advances in structural virology and immunoinformatics have enabled the identification of conserved immunodominant antigens from both mature virion and extracellular virion forms, supporting the development of multivalent antigen combinations capable of inducing broad neutralizing antibody (nAb) responses. Emerging delivery technologies including mRNA-lipid nanoparticles, viral vectors, and self-assembling protein nanoparticles offer rapid scalability, enhanced immunogenicity, and improved safety compared with conventional live-attenuated vaccines. Addressing antigenic evolution, vaccine supply limitations, and population-specific immune variability will be crucial for optimizing vaccine effectiveness. This review synthesizes current evidence on antigen design, vaccine delivery platforms, and immunological correlates of protection to outline a framework for next-generation mpox vaccines. Precision vaccinology therefore represents a transformative strategy for developing durable, clade-specific mpox vaccines and strengthening preparedness against future orthopoxvirus outbreaks worldwide.

## 1. Introduction

Mpox has evolved from a sporadic zoonosis into a significant global public health crisis [1]. Traditionally endemic to the tropical rainforests of Central and West Africa, the monkeypox virus (MPXV) triggered an unprecedented international outbreak in May 2022, primarily driven by clade IIb transmission within human-to-human networks [2]. By August 2024, the World Health Organization declared a second Public Health Emergency of International Concern following the emergence of more virulent clade Ib strains in the Democratic Republic of the Congo, which exhibit higher mortality rates exceeding 10% [3]. This expansion highlights a critical vulnerability in population immunity following the cessation of routine smallpox vaccination in 1980 [4]. Consequently, the median age of infection has shifted towards younger, immunologically naïve adults who lack cross-protective orthopoxviruses (OPXVs) immunity [5].

Current preventive strategies rely on repurposed smallpox vaccines based on the vaccinia virus (VACV) due to high genetic homology [6,7]. While first- and second-generation vaccines like ACAM2000 are effective, they are associated with severe adverse events such as myopericarditis and are contraindicated for immunocompromised populations [4,8]. The third-generation, non-replicating MVA-BN vaccine (JYNNEOS) offers a superior safety profile but faces logistical hurdles and limited supply [4,9]. Real-world data indicate that JYNNEOS effectiveness ranges between 36% and 86%, with evidence that neutralizing antibodies (nAbs) are relatively low and wane significantly within 12 months [7,10]. Furthermore, current vaccines primarily target VACV-specific epitopes, which may not provide optimal protection against divergent MPXV clades [5].

**Figure 1:**
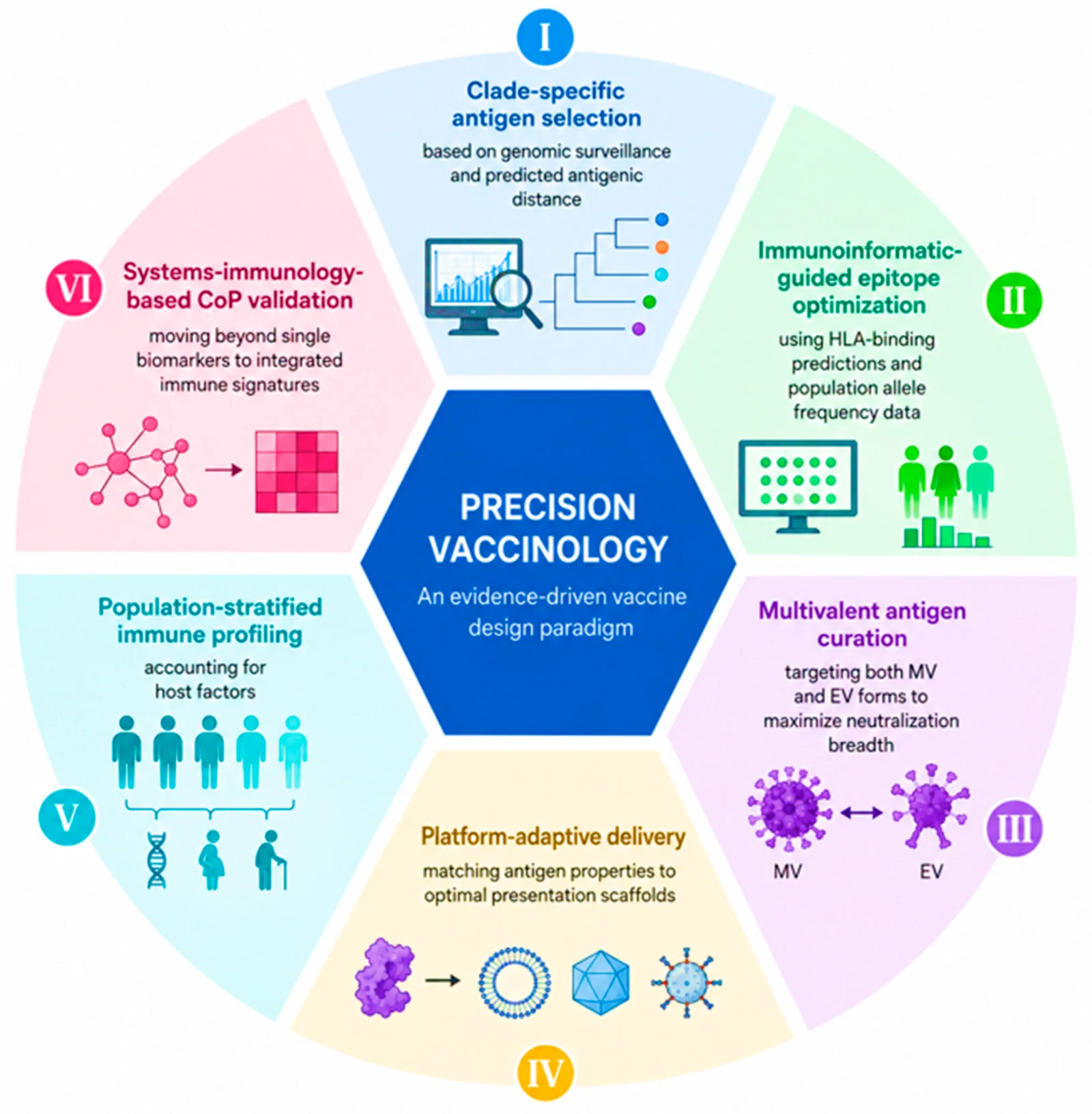
Conceptual framework for precision vaccinology. This image was generated by ChatGPT version GPT-5.6 Luna.

Precision vaccinology represents a paradigm shift toward designing mpox-specific interventions through immunoinformatics and rational antigen selection [11]. In the context of this review, precision vaccinology refers to an evidence-driven vaccine design paradigm characterized by VI integrated components (Figure 1) [12]: (I) clade-specific antigen selection based on genomic surveillance and predicted antigenic distance; (II) immunoinformatic-guided epitope optimization using HLA-binding predictions and population allele frequency data; (III) multivalent antigen curation targeting both MV and EV forms to maximize neutralization breadth; (IV) platform-adaptive delivery matching antigen properties to optimal presentation scaffolds; (V) population-stratified immune profiling accounting for host factors; and (VI) systems-immunology-based CoP validation moving beyond single biomarkers to integrated immune signatures (Figure 1) [12]. Figure 1 summarizes the six operational components of precision vaccinology. Each component represents a measurable stage within the vaccine-development pipeline. Clade-specific antigen selection is implemented through genomic surveillance and antigenic-distance analyses; immunoinformatics-guided optimization through HLA-binding prediction and population-coverage analyses; multivalent antigen curation by evaluating neutralization breadth against both MV and EV antigens; platform-adaptive delivery by matching antigen properties with appropriate delivery technologies; population-stratified immune profiling by assessing immune responses across different demographic and immunological groups; and systems-immunology-based CoP validation through integration of humoral, cellular, and functional immune biomarkers.

This approach focuses on identifying minimal protective antigen combinations from both the mature virion (MV) and extracellular enveloped virion (EV) forms such as M1, A29, B6, and A35 to achieve high-titer neutralizing responses [13,14]. Next-generation delivery platforms, including mRNA-lipid nanoparticles and self-assembling protein nanoparticles, offer the flexibility and rapid scalability required to address microevolutionary changes in the viral genome [4]. Moreover, precision strategies aim to establish multifaceted correlates of protection [15] by utilizing systems immunology to understand heterologous immune responses across diverse populations [16]. This ensures that future vaccines are tailored to specific clades rather than relying solely on vaccinia cross-reactivity.

**Figure 2:**
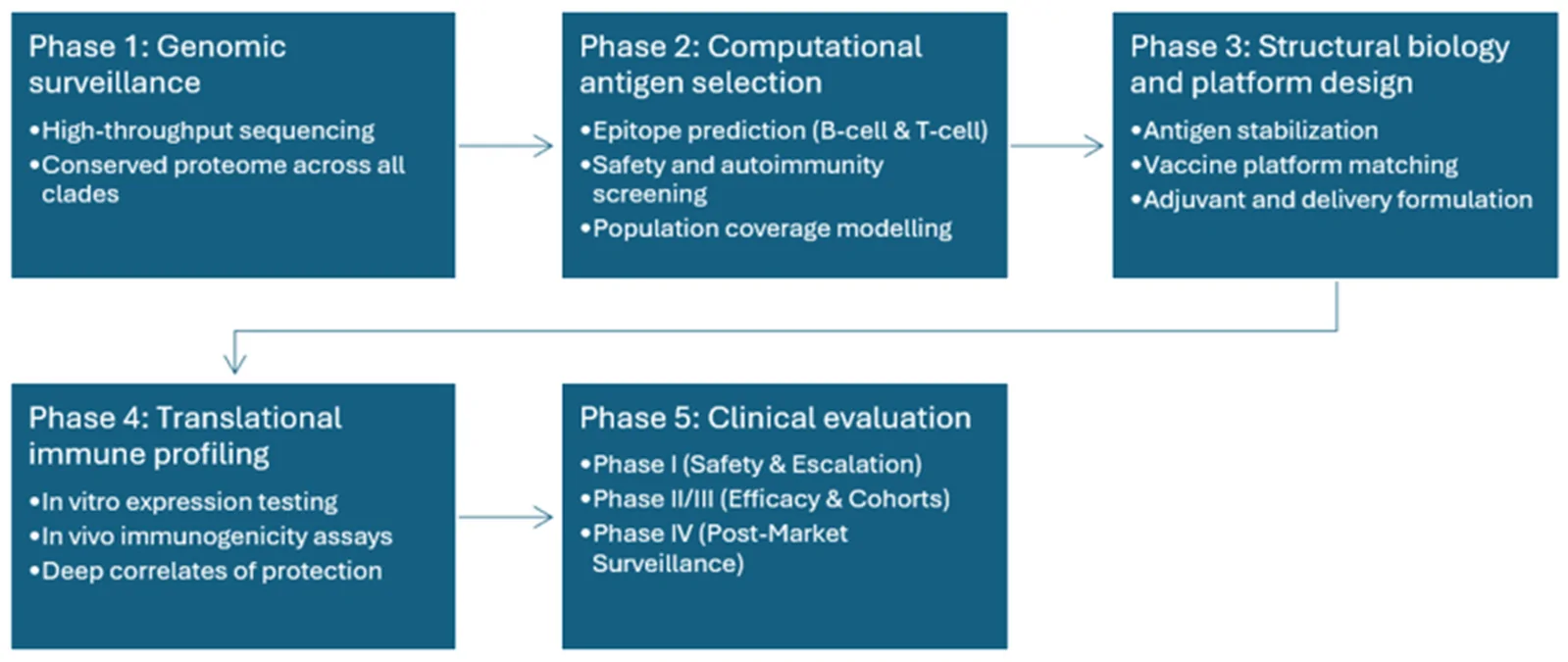
A Precision-Vaccinology Pipeline.

Precision vaccinology integrates genomic surveillance, computational antigen discovery, rational vaccine engineering, immunological profiling, and clinical evaluation into a unified framework for the development of next-generation vaccines (Figure 2) [15,17]. The process begins with genomic surveillance, where next-generation sequencing and third-generation sequencing technologies, such as Oxford Nanopore and PacBio, are used to rapidly characterize pathogen genomes and monitor their evolution. Comparative analyses of global genomic datasets enable the identification of conserved protein targets while excluding hypervariable regions, thereby improving the potential for broad and durable protection against emerging variants. Subsequently, reverse vaccinology and structural immunology approaches are employed to predict immunogenic B-cell and T-cell epitopes using machine-learning algorithms, while screening for potential cross-reactivity with human proteins and optimizing coverage across diverse human leukocyte antigen (HLA) alleles to ensure population-wide effectiveness. Selected antigens are then structurally optimized using computational modeling and experimental structural data to preserve highly immunogenic conformations and are incorporated into appropriate delivery platforms, including mRNA vaccines, viral vectors, recombinant protein subunits, or virus-like particles (VLPs). Advanced delivery systems, such as lipid nanoparticles (LNPs) and polymeric carriers, further enhance antigen stability, cellular uptake, and immune activation. The resulting vaccine candidates undergo rigorous preclinical validation through in vitro expression studies and in vivo immunogenicity assessments, where humoral responses are evaluated using enzyme-linked immunosorbent assays (ELISA) and neutralization assays, while cellular immunity is characterized through flow cytometry and enzyme-linked immunospot (ELISpot) analyses. Promising candidates subsequently advance through Phase I, II, and III clinical trials to assess safety, immunogenicity, optimal dosing regimens, and protective efficacy in increasingly larger populations. Following regulatory approval, continuous post-marketing surveillance evaluates long-term effectiveness, monitors rare adverse events, and assesses protection against newly emerging variants. Collectively, this precision vaccinology pipeline enables the rapid, data-driven, and adaptive development of vaccines with improved safety, efficacy, and resilience against evolving infectious diseases. 

Unlike previous reviews that primarily discuss mpox vaccine platforms, orthopoxvirus immunity, or individual aspects of vaccine development, this review provides an integrated precision vaccinology perspective by combining advances in rational antigen selection, reverse vaccinology, immunoinformatics, structural vaccinology, vaccine delivery platform engineering, and immune correlates of protection. By synthesizing these complementary approaches within a single framework, this review highlights current progress, existing challenges, and future directions for the rational design of next-generation mpox vaccines.

## 2. Survey Methodology

Relevant literature was identified through searches of PubMed and Google Scholar using the following keywords: monkeypox virus; vaccine development; rational antigen design; immunogen design; structural vaccinology; reverse vaccinology; epitope mapping; mRNA vaccines; DNA vaccines; viral vector vaccines; recombinant protein vaccines; nanoparticle vaccines; virus-like particles (VLPs); immune correlates of protection; neutralizing antibodies; humoral immunity; cellular immunity; and vaccine immunogenicity. No strict timeframe was imposed, but priority was placed on recent advancements. Advanced searches were refined using Boolean operators tailored to each database. The principal Boolean search string employed was: (“Mpox” OR “monkeypox” OR “monkeypox virus” OR MPXV OR orthopoxvirus) AND (“vaccine” OR vaccinology) AND (“antigen design” OR “immunogen design” OR “rational vaccine design” OR “reverse vaccinology” OR “epitope mapping”) AND (“mRNA vaccine” OR “RNA vaccine” OR “DNA vaccine” OR “viral vector vaccine” OR “subunit vaccine” OR “recombinant protein vaccine” OR “nanoparticle vaccine” OR “virus-like particle”) AND (“immune correlates of protection” OR “correlates of immunity” OR “protective immunity” OR “neutralizing antibodies” OR “humoral immunity” OR “cellular immunity” OR “T-cell response” OR “B-cell response”) AND (“animal model” OR “challenge study” OR immunogenicity OR efficacy OR “systems vaccinology” OR “immune profiling”) (accessed on 2 January 2026). In addition, the inclusion criteria were limited to English-language articles addressing mpox or related orthopoxvirus vaccine development, immunological mechanisms of protection, vaccine platforms, or preclinical and clinical evaluation. Studies lacking relevance to vaccine design, immune responses, or protective correlates were excluded. Findings were synthesized to provide a comprehensive assessment of precision vaccinology approaches for mpox, emphasizing rational antigen design, next-generation vaccine technologies, immune correlates of protection, current knowledge gaps, and future directions for vaccine development.

## 3. Mpox Virology, Structural Proteins, and Host Cell Entry

The MPXV is a complex, large, enveloped double-stranded DNA (dsDNA) virus with a linear genome ranging from approximately 190 to 211 kilobases (kb) [18]. The genomic architecture is characterized by a highly conserved central core region, typically spanning the middle 100 kb, which encodes essential genes required for transcription, DNA replication, and virion assembly [19]. This core is flanked by hypervariable terminal regions containing inverted terminal repeats (ITRs) of approximately 10 kb [19]. These terminal regions primarily encode accessory proteins involved in host range determination, immunomodulation, and virulence, which are often divergent between OPXVs species [19]. The MPXV genome encompasses between 181 and over 200 non-overlapping open reading frames (ORFs) [11]. Of these identified ORFs, more than 30 encode structural proteins that constitute the mature infectious virion [20]. Conversely, non-structural proteins are expressed during the early stages of the viral life cycle within the host cell cytoplasm, where the entirety of MPXV replication occurs [21].

The MPXV virion is a brick- or oval-shaped particle measuring approximately 200–300 nm in diameter [18]. Its internal structure consists of a biconcave nucleoprotein core containing the viral genome, flanked by two lateral bodies of unknown function, all of which are enclosed within a lipoprotein envelope. The viral life cycle produces two antigenically distinct infectious forms: the intracellular mature virion (IMV), also termed the mature virion (MV), and the extracellular enveloped virion (EEV), or extracellular virion (EV) [11]. MVs possess a single membrane and are released only upon host cell lysis, serving as the primary form for inter-host transmission. EVs are formed when MVs are wrapped in additional Golgi-derived membranes and exit the cell via exocytosis; these particles possess an extra lipid envelope that facilitates efficient, rapid cell-to-cell spread and long-range dissemination within the host while evading initial immune detection [21].

The structural proteins of MPXV are the primary targets for the host’s adaptive immune response, particularly for the induction of nAbs. Critical targets for neutralizing the MV form include M1R, A29L, H3L, E8L, and A30L [22]. M1R is a myristylated surface protein essential for viral entry and membrane fusion, while A29L and E8L facilitate initial attachment to the host cell [23]. Primary targets on the EV form include A35R and B6R [13]. B6R functions as a complement control protein and is required for efficient viral spread, while A35R is vital for the formation of actin-containing microvilli that drives cell-to-cell transmission [23,24].

Viral entry into host cells is a coordinated multi-step process involving attachment, hemifusion, and core penetration. MV particles initiate infection by binding to anionic host cell surface glycosaminoglycans. Specifically, the A29L protein mediates interaction with heparan sulfate, while the E8L protein binds to chondroitin sulfate. Attachment is followed by membrane fusion, a process cooperatively mediated by a conserved entry-fusion complex (EFC) consisting of at least 8 to 11 proteins, including A21L, A28L, L1R, and G2R. The M1R protein is particularly critical for this fusion step, and its blockade by antibodies prevents the release of the viral core into the host cytoplasm (Table 1) [12,23].

The complexity of the MPXV proteome and the existence of two infectious forms necessitate a multivalent approach to antigen design. Precision vaccinology focuses on selecting a “tailored combination” of immunodominant surface proteins from both the MV and EV forms to achieve high-titer nAb responses and comprehensive protection. Systematic screening has identified 12 key antigens including A21, A29, M1, H3, A35, and B6 that vary in their neutralizing immunogenicity [3,22,25,26]. While targeting a single antigen such as M1R can elicit potent neutralizing antibody responses, monovalent strategies may be more susceptible to immune escape if amino acid substitutions occur within dominant neutralizing epitopes. For example, the experimentally characterized D35N substitution in M1R abolishes binding of the potent neutralizing antibody 7D11, highlighting the potential vulnerability of single-epitope targeting approaches [27,28,29]. Consequently, multivalent antigen formulations have been explored to broaden immune coverage and enhance protection. For example, a recombinant vaccine comprising the MPXV structural proteins A29, M1, A35 and B6 induced strong humoral and cellular immune response in BALB/c mice, characterized by elevated neutralizing antibody titers, increased IFN-γ production, and enhanced Th1-mediated immunity [30]. Vaccinated mice also showed reduced viral replication and pathological damage following challenge with a 2022 MPXV mutant strain [20]. These preclinical models have demonstrated enhanced protective efficacy and broader immune response suggesting that incorporating multiple antigens may improve protection against infection and limit systemic viral dissemination, although the extent to which such approaches confer durable or near-sterilizing immunity in humans remains to be established.

## 4. Immunological Landscape of Mpox Infection

The host immune response to MPXV is characterized by a coordinated transition from rapid, non-specific innate mechanisms to highly specialized adaptive defenses [5]. Natural MPXV infection elicits a robust immune response capable of managing the disease through the provocation of both arms of the immune system. Innate immune cells, including natural killer (NK) cells and monocytes, initiate the body’s primary defense within days of viral invasion by producing type I interferons (IFNs) and proinflammatory cytokines. This is followed by the maturation of adaptive immunity, involving robust antibody production and T-cell expansion, which typically peaks several weeks post-infection or vaccination. While the innate response acts to limit initial viral replication and spread, the adaptive response, characterized by the development of long-lived memory B and T cells, provides the basis for durable, cross-protective immunity against subsequent orthopoxvirus challenges [31].

### 4.1. Innate Immunity: Pattern Recognition and Cytokine Signaling

The innate immune system recognizes MPXV through pattern recognition receptors (PRRs) that distinguish pathogen-associated molecular patterns (PAMPs) [32]. Toll-like receptors (TLRs) play a central role in this sensing process: TLR3 detects viral double-stranded RNA (dsRNA), whereas TLR9 identifies unmethylated CpG DNA sequences characteristic of the viral genome [33]. Additionally, TLR2 is involved in activating NK cells and promoting the differentiation of memory cells into CD8^+^ T cells [34]. The activation of these receptors primarily utilizes the MyD88-dependent pathway to trigger NF-κB and MAPK signaling, resulting in the production of type I IFNs (IFN-α/β) and essential proinflammatory cytokines such as IL-6, TNF-α, and IL-12 [5]. Furthermore, viral dsRNA activates protein kinase R (PKR), which phosphorylates the eukaryotic translation initiation factor-2a (eIF2α), leading to translational arrest and the induction of host cell death to curtail viral synthesis. NK cells are also critical effectors, expanding significantly in the blood and lymph nodes post-infection to destroy infected cells via the release of perforin and granzymes [35,36].

### 4.2. Immune Evasion Strategies

Despite these robust defenses, MPXV employs a sophisticated array of proteins to evade host detection and signaling [37]. The virus produces the B16 protein (an ortholog of VACV B19), which functions as a secreted decoy receptor that binds type I IFNs, preventing them from interacting with host receptors and blocking downstream antiviral gene expression. To disrupt intracellular sensing, the viral F3L protein binds to dsRNA, thereby preventing the activation of the PKR pathway and reducing IFN production [38]. The MPXV p2 protein further inhibits the innate response by competitively blocking the nuclear translocation of interferon regulatory factor 3 (IRF3) [5]. Additionally, MPXV evades cellular immunity through the OMCP protein, a soluble ligand that binds to the NKG2D receptor on NK cells and CD8^+^ T cells, suppressing their cytolytic activity and ability to recognize infected cells [18]. The virus also produces cytokine decoys, such as BR-209, which neutralizes IL-1β, and CrmB that antagonizes TNF-α signaling [39].

### 4.3. Adaptive Immunity: T-Cell Responses and Memory Formation

Adaptive immunity is essential for the ultimate clearance of MPXV and the establishment of long-term protection [40]. MPXV infection activates CD4^+^ and CD8^+^ T cells, which drive a Th1-biased response [20]. CD4^+^ helper T cells facilitate B-cell maturation and the production of high-affinity nAbs, while T follicular helper (Tfh) cells support the germinal center reactions necessary for affinity maturation [41]. CD8^+^ cytotoxic T lymphocytes (CTLs) are crucial for eliminating viral reservoirs by recognizing MHC-I-presented viral peptides and executing direct antiviral effects. These T cells produce a range of inflammatory mediators, including IFN-γ, TNF-α, IL-2, and IL-8. Notably, MPXV-specific effector memory T cells (Tem) and tissue-resident memory cells are generated post-infection, with CD8^+^ memory cells capable of persisting for more than four decades after exposure to orthopoxvirus antigens [22]. This long-lived cellular memory can provide cross-reactive protection even when circulating nAb titers have waned below detectable thresholds. However, MPXV can interfere with this arm by using its M2 protein to bind B7 molecules on antigen-presenting cells, thereby disrupting the B7-CD28 costimulatory interaction and inhibiting T-cell activation [42]. Therefore, cellular immune parameters, including CD4^+^ T-cell help, CD8^+^ T-cell cytotoxicity, and memory T-cell formation, should be considered alongside neutralizing antibodies as complementary immune correlates of protection for evaluating long-term mpox vaccine efficacy (Table 2).

In conclusion, the structural complexity of MPXV and its reliance on two antigenically distinct infectious forms MVs for inter-host transmission and EVs for intra-host spread necessitate moving beyond single antigen designs. Incorporating a “tailored combination” of immunodominant surface proteins (such as M1, A29, B6, and A35) into multivalent formulations offers the most viable path to prevent both viral entry and cellular dissemination. Preclinical success with these multi-antigen strategies underscores their potential to overcome viral point mutations and provide broader, more resilient protective immunity.

## 5. Humoral Immune Responses and Correlates of Protection

MPXV infection and vaccination trigger a comprehensive humoral response involving IgM, IgG, and IgA isotypes [45]. In natural infection, high IgG and IgA levels correlate with quicker viral clearance, though these isotypes appear 3–5 days later in individuals living with HIV [5]. For the third-generation vaccine MVA-BN (JYNNEOS), IgG levels in naïve persons typically peak approximately 8 weeks after the initial dose and wane significantly thereafter, with an estimated antibody half-life of 108 days [16,46]. In contrast, next-generation mRNA platforms demonstrate faster kinetics, showing robust IgG increases within 14–21 days after a booster dose [47]. Precision vaccinology utilizing multivalent mRNA vaccines highlights a Th1-dependent skewing toward IgG2a/c subclasses in murine models [48,49]. These subclasses are critical for high-affinity interaction with Fcγ receptors, facilitating more effective viral containment than the broader, less specific subclass profile often induced by traditional live-attenuated platforms.

Neutralizing antibody responses are the cornerstone of orthopoxvirus immunity, yet clinical evidence for MVA-BN reveals moderate potency. In naïve individuals, JYNNEOS elicits modest MPXV nAb titers, with a peak geometric mean PRNT50 of approximately 1:35 shortly after the second dose [10]. These levels are consistently lower than VACV-specific titers and wane rapidly, often falling below the limit of detection within 12 months. Conversely, smallpox-experienced individuals mount robust anamnestic responses, with titers nearly 10 times higher than naïve vaccinees after MVA-BN boosting [31]. Multivalent mRNA candidates indicate significantly higher potency; for example, BNT166 induced nAb titers of ~7947, while AR-MPXV5 reached 1:13,383 [20]. Efficacy is heavily influenced by antigenic distance; as the genetic similarity between the vaccine strain and circulating MPXV clades decreases, cross-neutralization potency declines [50]. This underscores the necessity of designing clade-specific vaccines rather than relying solely on vaccinia cross-reactivity.

Direct neutralization is frequently complemented by non-neutralizing humoral mechanisms, which are vital when circulating nAb titers are suboptimal. Fc-mediated effector functions include antibody-dependent cellular cytotoxicity (ADCC), antibody-dependent cellular phagocytosis (ADCP), antibody-dependent neutrophil phagocytosis (ADNP), and antibody-dependent complement deposition (ADCD) [16]. Evidence from passive transfer experiments in primates confirms that antibodies are necessary and sufficient for protection against severe disease, as serum transfer alone can prevent mortality in immunodeficient models [9]. Furthermore, the complement system is an essential cofactor for functional immunity; the addition of complement in vitro enhances neutralizing potency by 2- to 80-fold depending on the host species [51]. This is particularly critical for the extracellular EV, as anti-EV antibodies require complement activation to disrupt the outer lipid membrane and expose the internal MV for clearance.

The quality of the humoral response is associated with specific cytokine signatures that modulate B-cell activity. Protective responses are linked to a Th1-biased humoral signature, involving elevated levels of IFN-γ, TNF-α, IL-6, and IL-2 [49]. Precision mRNA platforms significantly upregulate these cytokines, mirroring the profiles seen in natural infection. Immunological simulations demonstrate that peak levels of IFN-γ, TGF-β, and IL-10 occur after repetitive antigen exposure, with IL-2 serving as a critical signal for macrophage activation [52]. Furthermore, Th17-associated cytokines like IL-17A and IL-22 are induced in precision vaccine models, potentially assisting in memory B-cell maturation and sustained antibody production [6]. Conversely, a dominant Th2 response is associated with increased disease severity, highlighting the importance of precision platforms in skewing immunity toward protective mediators [5].

Identifying a validated CoP remains the foremost challenge for precision vaccinology. While nAb titers are the most biologically plausible candidate, breakthrough infections have been documented in individuals with high circulating titers, indicating that antibody magnitude alone does not fully predict immunity [16]. Furthermore, nAb responses wane faster in children and people living with HIV (PLHIV), necessitating a full two-dose MVA-BN schedule to maximize seroconversion. Precision frameworks must therefore move toward an integrated immune profile that considers peak nAb titers, IgG subclass functionality, and Fc-mediated mechanisms alongside cellular memory. Establishing these correlates through longitudinal studies is essential for evaluating vaccine durability and informing global health policy. This integration of systems immunology will ensure that next-generation vaccines provide the robust, long-term protection necessary to contain emerging MPXV clades.

Although neutralizing antibody titers are a key correlate of orthopoxvirus immunity, they alone do not fully predict protection. Effective humoral immunity also depends on Th1-driven IgG subclass responses that mediate Fc effector functions and complement activation. Therefore, next-generation vaccines should elicit both potent neutralizing antibodies and durable humoral and cellular immune responses.

## 6. Pre-Existing Immunity and Cross-Reactivity

The decline of population immunity to OPXVs is a direct consequence of the cessation of routine smallpox vaccination in 1980 [21]. Younger cohorts born after this period lack the cross-protective immunity conferred by VACV, making them significantly more vulnerable to MPXV. Observational data indicate that historical smallpox vaccination remains approximately 85% effective against mpox [1]. Clinical studies demonstrate that individuals vaccinated decades ago retain detectable anti-VACV and anti-MPXV IgG, contributing to a 5.2-fold reduced risk of infection and significantly milder disease presentation compared to unvaccinated counterparts.

The molecular basis for this protection lies in the high antigenic homology (90–99%) shared among OPXVs [53]. Surface proteins such as M1, A29, B6, and A35 exhibit 94–98% sequence similarity between VACV and MPXV, allowing for broad cross-reactivity. In smallpox-experienced populations, a single booster dose of the third-generation MVA-BN (JYNNEOS) vaccine triggers a rapid recall of memory B cells, resulting in a potent anamnestic response [16]. This results in nAb titers nearly 10 times higher than those observed in vaccine-naïve individuals. However, recent research underscores that cross-neutralization potency is an inverse function of “antigenic distance”. While antibodies raised against VACV cross-react with MPXV orthologs, the neutralizing capacity against MPXV is consistently lower than against more closely related species like cowpox. This is partly due to discrete amino acid mutations in recent MPXV clades, particularly near neutralizing sites on proteins like A35R and E8L, which may facilitate immune evasion [41].

Beyond humoral responses, pre-existing cellular immunity is critical for long-term protection. CD8+ CTL memory remains functional for over four decades post-VACV exposure, with some studies detecting cross-reactive T-cell memory up to 80 years post-vaccination [50]. Approximately 80% of T-cell epitopes are conserved between VACV and MPXV, enabling memory T cells to rapidly respond to heterologous MPXV exposure even when circulating nAb titers have waned [16]. This enduring cellular memory, particularly Th1-biased effector memory cells, is vital for viral clearance and limiting systemic spread [22]. Consequently, historically vaccinated individuals exhibit reduced incidence of severe symptoms, fewer lesions, and lower hospitalization rates during breakthrough infections [43]. Integrating these cross-reactive signatures is essential for precision antigen design to improve vaccine breadth.

Overall, routine smallpox vaccination has demonstrated the long-term cross-protective potential of orthopoxvirus immunity. However, mutations in circulating MPXV clades have increased the antigenic distance from traditional vaccinia strains, resulting in reduced cross-neutralization. Addressing this gap will require antigen design strategies that preserve conserved B-cell and T-cell epitopes while accounting for clade-specific genetic variation.

## 7. Antigen Design Strategies for Precision Mpox Vaccines

### 7.1. Protective Antigen Identification

Precision antigen design for MPXV necessitates a shift from whole-virus vaccinia-based models toward the identification of discrete, immunodominant surface proteins. The MPXV genome, which spans approximately 197 kilobases and encodes over 190 ORFs, presents a complex antigenic landscape. Protective immunity is primarily directed at the two distinct infectious forms: MV/IMV, which mediates inter-host transmission, and EV/EEV, which facilitates intra-host spread. Systematic screenings have identified approximately 12 key antigens including A21, A29, M1, H3, A17, A30, A28, H2, I2, and G2 from MVs, and A35 and B6 from EVs that induce varying levels of neutralizing immunogenicity [22]. Among these, M1 (the L1 ortholog) and A29 (the A27 ortholog) are critical for viral entry and attachment, while B6 and A35 facilitate complement control and actin-mediated cell-to-cell spread, respectively. These proteins exhibit high conservation across OPXVs, generally exceeding 93% amino acid identity, which provides the molecular basis for cross-protective precision vaccines.

### 7.2. Reverse Vaccinology and Immunoinformatics for Rational Antigen Discovery and Vaccine Design

The integration of reverse vaccinology and immunoinformatics has transformed vaccine discovery by replacing empirical antigen screening with genome-wide computational analyses, thereby improving the efficiency of antigen identification and candidate prioritization [54]. Advances in whole-exome sequencing and RNA sequencing (RNA-seq) have further supported rational vaccine design by identifying candidate antigens and confirming their expression in relevant biological contexts [54]. For infectious diseases, reverse vaccinology integrates genomic, proteomic, and comparative genomic data to prioritize proteins based on characteristics such as surface or extracellular localization, sequence conservation, limited homology to host proteins, and favorable physicochemical properties [55,56]. Modern pipelines increasingly incorporate machine learning and deep learning approaches, including Vaxign-ML and Vaxign-DL, to improve antigen prioritization, while pan-genomic analyses identify conserved antigens across multiple strains to support broad-spectrum vaccine development [55,56]. Nevertheless, these computational approaches primarily facilitate candidate prioritization and require experimental validation to confirm immunogenicity and protective efficacy.

Following antigen prioritization, immunoinformatics provides an integrated framework for identifying immunogenic determinants and designing vaccine constructs capable of eliciting both humoral and cellular immune responses. Current workflows combine multiple computational tools to predict cytotoxic T-lymphocyte (CTL), helper T-lymphocyte (HTL), and B-cell epitopes while simultaneously evaluating antigenicity, immunogenicity, allergenicity, toxicity, conservancy, physicochemical properties, and population coverage to prioritize promising candidates [57,58]. Sequence-based tools such as NetMHC, NetMHCII, and ABCpred are widely used to predict peptide binding and linear B-cell epitopes. More recently, transformer-based models, including EpiBERTope, have improved the prediction of conformational B-cell epitopes by capturing contextual sequence information beyond conventional motif-based approaches [57,58].

Structural vaccinology has further enhanced these workflows through AI-based protein structure prediction platforms such as AlphaFold2, AlphaFold3, and RoseTTAFold [59]. These tools generate high-confidence structural models that enable the assessment of solvent accessibility, epitope exposure, conformational stability, and mutation-induced structural changes, even in the absence of experimentally determined [60,61]. Predicted structures can subsequently be integrated with molecular docking algorithms, such as HDOCK, and molecular dynamics simulations to evaluate interactions between vaccine candidates and immune receptors, including Toll-like receptors (TLRs), major histocompatibility complex (MHC) molecules, and T-cell receptors [62]. These structural analyses provide complementary evidence to support candidate prioritization rather than definitive proof of immunogenicity. Furthermore, advances in precision structural vaccinology have expanded structure-guided antigen engineering approaches, enabling the rational optimization of antigen conformation and epitope presentation to improve vaccine immunogenicity [63]. Strategies such as prefusion antigen stabilization, structure-guided epitope optimization, and computational immunogen design have demonstrated significant potential for enhancing antigen stability and eliciting stronger neutralizing immune responses across multiple viral pathogens [63]. Although these strategies have been most extensively applied to viruses such as SARS-CoV-2 and RSV, they provide a valuable conceptual framework for the future development of next-generation MPXV vaccines [63].

Immunoinformatics has also facilitated the rational design of multi-epitope vaccines by combining conserved CTL, HTL, and B-cell epitopes using optimized linker sequences, including EAAAK, GPGPG, AAY, and KK, together with molecular adjuvants such as PADRE peptides or β-defensins to improve structural organization and immunogenic potential [64]. In parallel, sequence optimization tools such as LinearDesign have been incorporated into mRNA vaccine development to enhance transcript stability and translational efficiency through codon optimization and secondary structure refinement [65]. Collectively, reverse vaccinology, immunoinformatics, structural biology, and artificial intelligence have accelerated vaccine candidate discovery and construct optimization. However, these computational approaches generate predictive evidence and should be considered complementary to, rather than substitutes for, experimental immunological validation.

Despite significant advances, important biological and technical limitations continue to restrict the translation of computational predictions into clinically effective vaccines. Most prediction algorithms estimate peptide–MHC binding affinity rather than the full complexity of antigen processing, peptide presentation, and immune recognition, resulting in only limited correspondence between predicted and naturally presented epitopes [66]. Prediction accuracy remains particularly challenging for MHC class II epitopes because of extensive HLA polymorphism and limited experimentally validated datasets [67]. Similarly, sequence-based methods often inadequately predict conformational B-cell epitopes, which depend on native protein structure. Additional challenges include antigen processing complexity, HLA diversity across populations, and pathogen evolution, all of which influence vaccine effectiveness and population coverage [64]. Therefore, computational approaches should be regarded as candidate prioritization tools, with rigorous experimental validation through immunological assays, structural studies, animal models, and clinical investigations remaining essential to confirm immunogenicity, protective efficacy, and safety. Currently, computational antigen-selection approaches, including reverse vaccinology, immunoinformatics, and machine learning, remain primarily predictive tools for mpox vaccine development. Although these methods have accelerated antigen discovery and candidate prioritization, not computationally designed mpox vaccine candidate has yet achieved clinical validation, highlighting the continued requirement for experimental and clinical evaluation.

### 7.3. Multi-Antigen and Multi-Epitope Vaccines

Evidence from preclinical models demonstrates that monovalent vaccines are often insufficient to provide complete protection against disease-related weight loss and systemic spread [48]. Precision strategies, therefore, emphasize “Mix-4” (M1, A29, B6, A35) or Mix-12 combinations to induce a more diversified and potent humoral response. The “Mix-4” antigen combination, consisting of M1, A29, B6, and A35, is favored because these antigens provide complementary immune targets across different stages of orthopoxvirus infection [68]. Unlike single-antigen approaches, Mix-4 includes antigens associated with both intracellular mature virion (IMV) and extracellular enveloped virion (EV) forms, enabling broader immune recognition and targeting of multiple viral processes [13].

M1 is a highly conserved IMV membrane protein that is abundantly expressed and structurally accessible on the virion surface, allowing antibody recognition that may interfere with viral entry and promote cross-reactive immunity among orthopoxviruses [23]. A29 is another IMV-associated protein involved in host cell attachment, and antibodies against A29 can neutralize infection by blocking early virion cell interactions [12]. In contrast, B6 and A35 are EV-associated antigens that contribute to extracellular viral dissemination, with antibodies against these proteins limiting viral spread and reducing cell-to-cell transmission [24]. The inclusion of both IMV- and EV-targeting antigens therefore provides a broader neutralization strategy compared with targeting a single viral form. However, multivalent formulations must also consider potential antigenic competition and immune interference, as highly immunogenic components may dominate antigen presentation or antibody responses, reducing responses against less immunodominant antigens [13]. Competition for antigen-presenting cells, T-cell help, or B-cell activation pathways may influence the magnitude and balance of responses generated against individual components [13]. Additionally, differences in antigen abundance, structural conformation, and epitope accessibility may affect the contribution of each antigen to overall protection [12]. Thus, the rationale for Mix-4 lies in its complementary antigenic coverage and potential to induce broader protective immunity rather than simply combining highly immunogenic proteins. Further experimental evaluation of antigen ratios, immune dominance, neutralizing antibody breadth, and protective efficacy remains necessary to determine whether these antigens provide additive or synergistic benefits in multivalent vaccine formulations.

In head-to-head mouse model studies, the Mix-12 group exhibited the highest titers of nAbs (NT50) which is 1195 ± 200 against MPXV [22]. While Mix-4 also induced robust titers that were superior to any single-antigen vaccine, they were significantly lower than those induced by Mix-12 which is 644 ± 75 [22]. This suggests that adding more antigens, even those with moderate or low individual neutralizing potential, results in a synergistic enhancement of the total humoral response. The results demonstrated that increasing antigen diversity by including antigens with high, moderate, and even low individual neutralizing potential leads to a more potent immune response. The authors explicitly suggest that this synergistic enhancement is due to addressing the polymorphism of monkeypox-neutralizing antibodies, emphasizing that the diversity of antibodies is a critical factor in providing potent protection against high-titer viral challenges.

Beyond antibodies, the integration of T-cell epitope-rich regions (MPX-EPs) can independently confer protection by targeting cellular immunity, essential for clearing infected cells and mitigating disease progression. Multivalent mRNA platforms, such as BNT166 and mRNA-1769, have utilized these combinations to achieve 100% survival and near-sterilizing immunity in lethal non-human primate and murine challenges [7].

### 7.4. Recombinant Protein, VLP, and Nanoparticle-Based Platforms

Advanced delivery platforms are being engineered to enhance the immunogenicity and stability of these tailored antigens. Platforms using self-assembling scaffolds like lumazine synthase (LuS), mi3, or ferritin present antigens in a high-density, multivalent format that mimics viral geometry [53]. These particles improve B-cell receptor cross-linking and enhance antigen uptake by dendritic cells (DCs) in lymph nodes. Unlike monovalent, mosaic nanoparticles can co-display multiple different antigens on a single scaffold, eliciting a broader and more balanced immune signature. Certain protein nanoparticles, such as AaLS-based constructs, demonstrate excellent thermostability, remaining stable after one week of storage at 37 °C, thereby reducing dependence on cold-chain logistics [53].

While precision vaccines offer superior safety profiles, avoiding the risks associated with replicating viruses, they face distinct challenges. Multivalent protein formulations can increase manufacturing complexity and cost, potentially leading to imbalanced antigen utilization. mRNA vaccines provide rapid scalability and adaptability but currently require ultra-cold storage, which limits equitable distribution in resource-constrained regions [4]. Furthermore, the microevolution of MPXV noted by over 50 single nucleotide polymorphisms (SNPs) in recent lineages poses a risk of neutralization escape, particularly for monovalent targets like M1 [2]. A critical research gap remains the lack of human clinical data specifically for clade I performance, as most efficacy is still inferred from vaccinia or clade IIb models. Future research must prioritize identifying multifaceted CoP and establishing the long-term durability of these next-generation designs in diverse human populations.

In conclusion, rational antigen design shifts vaccine development from whole-virus formulations to computationally optimized, multi-epitope constructs. Preclinical data demonstrate that multivalent combinations such as Mix-4 or Mix-12 displayed on self-assembling nanoparticles elicit an antibody response capable of neutralizing multiple viral morphologies. Although managing formulation and manufacturing complexity remains a challenge, these precise structural frameworks provide a foundation for achieving broad protection against diverse viral lineages.

## 8. Vaccine Delivery Platforms

The rapid global expansion of mpox has catalyzed a shift from traditional vaccinia-based models toward a diversified landscape of next-generation vaccine delivery platforms ranging from licensed orthopoxvirus vaccines with established clinical effectiveness to early-stage nucleic acid, viral vector, protein subunit, and nanoparticle candidates. Direct comparisons of immunogenicity across different vaccine platforms should be interpreted with caution. Reported neutralizing antibody (nAb) titers are often generated using different assay methodologies, including plaque reduction neutralization tests (PRNTs), pseudovirus neutralization assays, and binding antibody assays, each of which differs in sensitivity, specificity, and endpoint definition. Furthermore, studies vary in their use of animal models (e.g., mice, non-human primates, and humans), viral challenge strains, vaccination schedules, sampling time points, and immunological endpoints, all of which can influence the magnitude and kinetics of the measured immune responses. Consequently, reported nAb values should not be interpreted as direct head-to-head comparisons of vaccine performance but rather as study-specific indicators of immunogenicity. Standardization of immunological assays, study designs, and clinical endpoints will therefore be essential to enable meaningful comparisons across future mpox vaccine studies. Accordingly, vaccine strategies interpreted within an evidence hierarchy spanning clinical, early translational and preclinical stages (Table 3).

### 8.1. Highest Evidence Tier of Clinically Validated Vaccines

Live vaccinia-based vaccines (VACV) remain the only platforms with robust human evidence for protection against OPXV related infections. ACAM2000, a replication-competent VACV vaccine, confers strong protective immunity but is limited by safety concerns, including vaccine-associated myopericarditis, restricting its use in immunocompromised populations [8]. In the other hand, replication-deficient MVA-BN (JYNNEOS) offers a superior safety profile and is suitable for at-risk groups, though it generally induces lower neutralizing antibody responses in naïve recipients compared with replication-competent vaccines in naïve persons (Table 4) [70]. In additions, LC16m8 vaccine approved in Japan represents an intermediate attenuation strategy with favorable safety and immunogenicity profiles, but its evidence base remains geographically limited, but significantly has milder side effects compared to ACAM2000 [71].

### 8.2. Preclinical to Early Translational Evidence of Viral Vector Vaccines

Viral vector-based platforms aim to improve safety and antigen specificity by delivering specific MPXV antigens without the risks associated with whole-virus replication in humans. Avipoxvirus-based vectors such as fowlpox (FWPV) are of interest due to their restricted replication in avian species while remaining transcriptionally active in mammalian cells [76]. This biological property allows efficient antigen expression without productive infection in humans, thereby enhancing safety. A key advantage of FWPV is that it does not immunologically cross-react with mammalian poxviruses, allowing it to escape vector-generated neutralization in individuals with pre-existing smallpox vaccine immunity. Preclinical studies suggest that FWPV-based constructs can induce protective immunity against orthopoxvirus challenge in animal models, particularly when used in heterologous prime-boost regimens such as DNA priming followed by FWPV viral vector boosting [76]. However, these findings are primarily derived from murine or small-animal challenge models, and no human efficacy data are currently available. Furthermore, the impact of pre-existing immunity to vector components and the durability of protection remain unresolved.

### 8.3. Early Clinical and Preclinical Evidence of mRNA and DNA Vaccines

Nucleic acid-based vaccines have emerged as highly adaptable platforms enabling rapid antigen design and scalable cell-free manufacturing. mRNA vaccines formulated in lipid nanoparticles (LNPs) encode conserved orthopoxvirus surface antigens like M1R, A29L, A35R, and B6R [69]. Preclinical data show that multivalent mRNA candidates like BNT166 and mRNA-1769 induce nAb titers exceeding 1:13,000 in non-human primates, which are significantly higher than those elicited by MVA-BN in similar animal models. Furthermore, mRNA platforms can encode these antigens tandemly in a single molecule, simplifying the manufacturing process while ensuring a balanced immune response in vivo; however, extrapolating these animal immunogenicity magnitudes to predict confirmed human clinical protection requires extreme caution, as human clinical trial data are currently absent [2,3]. DNA-based vaccines offer high stability and simple manufacturing but generally exhibit lower immunogenicity in large animal models and humans compared to small rodents [76]. This reduced efficacy is largely attributed to inefficient cellular uptake and nuclear entry of plasmid DNA in vivo [77]. Although physical delivery enhancement strategies such as electroporation improve immune responses, clinical translation remains limited, positioning DNA vaccines primarily within early exploratory development [77]. These findings remain largely confined to preclinical studies, and no DNA-based mpox vaccine has advance to late-stage clinical efficacy trials, limiting their current translational maturity [78].

### 8.4. Preclinical Evidence with Translational Potential of Protein Subunit and Nanoparticle Vaccines

Protein subunit vaccines focus on a controlled set of pathogen components, offering high safety and stability. The immunogenicity of these vaccines is markedly improved by self-assembling nanoparticles (e.g., mi3, LuS, AaLS) [13], which display antigens in a high-density, multivalent format mimicking viral geometry. These platforms enhance B-cell receptor cross-linking and facilitate efficient uptake by dendritic cells in the lymph nodes. Preclinical studies have demonstrated that trivalent nanoparticles displaying M1, E8, and B6 antigens have demonstrated superior protective efficacy and enhanced viral clearance in respiratory tissues compared to monovalent protein cocktails within murine and rodent respiratory challenge models. This is because human mucosal transmission routes and respiratory immune networks differ substantially from rodents; these findings represent candidate mechanisms that remain unconfirmed in human clinical efficacy trials [3]. Similarly, recombinant protein cocktails containing antigens such as A29L, M1R, A35R, and B6R have shown protective efficacy in preclinical models, but their performance is strongly dependent on adjuvant selection and delivery formulation. An additional advantage of nanoparticle-based system is their improved physicochemical stability, with some platforms demonstrating thermostability at 37 °C for extended periods, potentially reducing cold-chain dependence [2,3]. Nevertheless, these technologies remain in the preclinical to early translational stage, and their clinical protective efficacy has not yet been established [13,21].

Mpox vaccine platforms exhibit distinct profiles across key translational and deployment parameters, including safety, immunogenicity, redesign speed, manufacturing scalability, cold-chain requirements, suitability for endemic settings, cost, reactogenicity, regulatory feasibility, and the induction of balanced immune responses against both mature virion (MV) and extracellular virion (EV) forms. Overall, next-generation platforms such as mRNA and nanoparticle-based vaccines offer greater design flexibility and strong immunogenic potential, whereas established platforms, including live attenuated and viral vector vaccines, provide robust and durable immunity but are constrained by safety considerations and deployment limitations (Table 5).

To summarize, the landscape of mpox countermeasures spans a translational spectrum from established live-attenuated vaccines to adaptable next-generation nucleic acid and nanoparticle candidates. Advanced platforms like mRNA-LNPs offer efficient redesign capabilities and strong preclinical immunogenicity, yet they present logistical distribution challenges in resource-constrained settings. Selecting and scaling an optimal delivery vehicle will require balancing vaccine safety, manufacturing costs, and deployment feasibility to meet the public health demands of endemic regions (Table 6).

## 9. Integrated Correlates of Protection for Precision Vaccinology

Despite substantial advances in mpox vaccine development, a validated human correlate of protection remains elusive. Current evidence suggests that protection is multifactorial, involving humoral, cellular, mucosal, and Fc-mediated immune responses, as well as clinical and virological outcomes. Consequently, multiple candidate biomarkers and surrogate endpoints are being investigated to better define protective immunity and guide the development and evaluation of next-generation mpox vaccines (Table 3).

### 9.1. Humoral and Cellular Immune Correlates

A definitive, validated immunological CoP for mpox in humans remains unestablished [80]. While nAb titers are considered a primary candidate for CoP due to their role in limiting viral entry and systemic spread in non-human primate (NHP) models, they provide an incomplete picture of immunity [3]. Precision vaccinology therefore emphasizes a composite profile that integrates humoral, cellular, and functional markers.

Evidence indicates that the virion form targeted influences protection: while MV antibodies block initial infection, extracellular EV antibodies show a significantly stronger correlation with reduced viral loads in respiratory tissues, such as the lungs and nasal turbinates [3]. Furthermore, the functionality of IgG subclasses is a critical correlate; Th1-biased IgG1 and IgG2a/c are associated with higher protective potency through effective Fcγ receptor engagement and complement-dependent deposition (ADCD). Beyond antibodies, T-cell responses are essential for clearance and durability. Th1-producing effector memory cells (Tem) can persist for decades post-exposure, providing a vital arm of protection that remains functional even when circulating nAb levels fall below detection thresholds.

### 9.2. Systems Immunology and Biomarker Discovery

Systems immunology utilizes high-dimensional profiling, such as single-cell RNA sequencing (scRNA-seq) and BCR/TCR sequencing, to uncover “immune signatures” of protection. Splenic transcriptional profiling post-vaccination reveals the significant expansion of plasma cells and neutrophils, which orchestrate antibody production and antigen presentation. BCR repertoire analysis has identified overrepresented genes notably *Ighv1-5* and *Igkv1-110* and specific hypervariable CDR3 motifs like “YAMDYW” and “GYYYFDY” as indicators of vaccine-induced clonal selection. Additionally, antibodies against A35R and H3L antigens have emerged as specific biomarkers for natural infection-driven immunity, as these often remain low following traditional MVA-BN vaccination [3].

### 9.3. Population-Specific Immune Response Variability

Precision strategies must account for host-specific factors that drive heterologous responses. PLHIV often mount significantly weaker peak nAb titers and exhibit faster antibody waning, with titers appearing 3 to 5 days later than in HIV-negative individuals [5]. This necessitates a full two-dose MVA-BN schedule regardless of prior smallpox vaccination status to maximize seroconversion. Demographic factors further influence immunity; some studies show males exhibit ~27% higher anti-MVA titers than females [5]. Children represent another population in whom immune responses to mpox vaccination remain insufficiently characterized. Current recommendations and expectations of vaccine-induced protection in pediatric populations are largely extrapolated from adult studies, historical smallpox vaccination data, and limited observational evidence [1,2,8]. Developmental differences in both innate and adaptive immunity, including ongoing maturation of T-cell responses and germinal center activity, may influence the magnitude, quality, and durability of vaccine-induced protection. Moreover, the safety, optimal dosing regimens, and long-term immunogenicity of next-generation mpox vaccines in children have not been comprehensively evaluated. Consequently, age-stratified immunogenicity studies and post-licensure surveillance are required to determine whether correlates of protection identified in adults are equally applicable to pediatric populations.

Similarly, immunocompromised populations, particularly PLHIV, warrant dedicated investigation. Although MVA-BN has demonstrated an acceptable safety profile in PLHIV, these individuals frequently exhibit delayed seroconversion, lower peak neutralizing antibody titers, and more rapid waning of humoral responses compared with HIV-negative individuals [5,46]. Completion of the full two-dose vaccination schedule therefore appears critical to maximize seroconversion and immunological memory irrespective of prior smallpox vaccination history [16,31]. These observations highlight the need to validate integrated CoPs across diverse host populations, as immune signatures predictive of protection may vary according to age, immune status, and prior orthopoxvirus exposure.

Age is also a major determinant, as individuals with historical smallpox exposure retain potent B-cell and T-cell memory that can be rapidly boosted by modern vaccines. Validating these integrated CoPs through longitudinal studies in diverse populations is essential for the design of durable, next-generation mpox vaccines.

**Figure 3:**
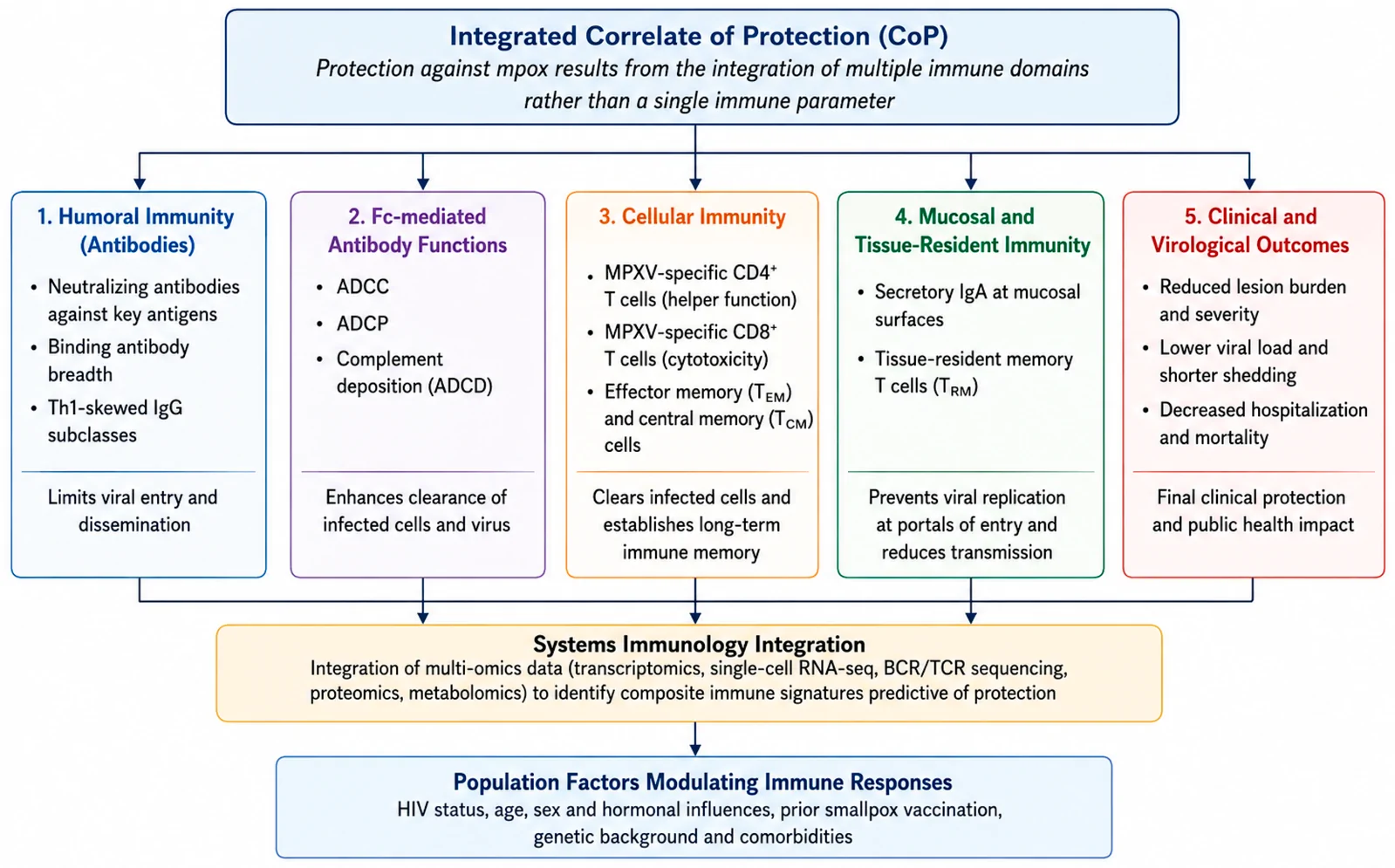
Proposed Working Model of Correlates of Protection for Precision Mpox Vaccinology.

Based on the current evidence summarized in this review, we propose an integrated working model in which protection against mpox is determined by the combined contribution of multiple immune domains rather than a single immunological marker (Figure 3). Humoral immunity, Fc-mediated antibody functions, cellular immunity, mucosal and tissue-resident immunity, and clinical and virological outcomes collectively form a multidimensional correlate of protection. Systems immunology approaches may facilitate the integration of these domains to identify composite immune signatures predictive of vaccine-mediated protection across diverse populations and vaccine platforms.

Ultimately, the lack of a validated human correlate of protection represents a key knowledge gap in mpox vaccinology. Moving forward, precision frameworks should utilize immunology systems to integrate neutralizing titers, Fc-effector functions, mucosal IgA, and memory T-cell signatures into composite biomarkers of protection. Validating these multi-domain immune signatures across diverse host demographics, including pediatric populations and individuals living with HIV, is essential for informing immunization policies and ensuring long-term clinical efficacy.

## 10. Current Challenges and Future Directions

Although MPXV is a dsDNA virus with a traditionally low substitution rate, recent outbreaks have revealed an accelerated evolutionary trajectory. The 2022 clade IIb lineage (B.1) contains more than 50 SNPs, a divergence significantly higher than anticipated [69]. A substantial proportion of these mutations (approximately 90%) are attributed to APOBEC3 cytosine deaminase activity, an innate host defense mechanism that preferentially modifies the MPXV genome. APOBEC3-mediated mutations have contributed substantially to the recent genomic diversification of MPXV and may alter predicted antigenic determinants. However, current evidence primarily supports genomic evolution rather than experimentally confirmed immune escape. The functional impact of these mutations on antibody neutralization, vaccine-induced protection, and clinical vaccine effectiveness remains insufficiently characterized and warrants further investigation [81,82].

Recent MPXV outbreaks have raised concerns regarding the impact of viral evolution on vaccine effectiveness. Although MPXV is a dsDNA virus with a relatively low evolutionary rate, the numerous mutations identified in clade IIb lineages suggest ongoing genomic diversification that may affect immunologically relevant epitopes. Nevertheless, continued genomic surveillance and functional studies assessing their effects on antibody neutralization and T-cell recognition remain essential. Multivalent vaccine strategies targeting multiple antigens may provide greater resilience against potential antigenic changes than monovalent approaches [2,69,81,82].

Several promising next-generation vaccine candidates discussed herein, including BNT166, mRNA-1769, and multivalent nanoparticle platforms, are currently supported by preclinical data from mouse and non-human primate (NHP) challenge models. While these models provide valuable proof-of-concept, they carry inherent limitations. Mouse models, though useful for initial immunogenicity screening, may not fully recapitulate human antibody Fc-effector functions or mucosal immune responses due to species-specific differences in Fcγ receptor architecture and complement pathways. NHP models more closely approximate human immunity but are limited by small sample sizes (typically *n* = 4–8 per group), high costs, and the use of lethal challenge endpoints that may not reflect natural transmission dynamics [83]. Furthermore, the vaccinia virus (VACV) challenge model used in many studies, while ethically necessary given MPXV biocontainment requirements, introduces antigenic distance that may overestimate or underestimate true MPXV protection [83]. A recent head-to-head comparison of mRNA-1769 and MVA-572 (a research-strain modified vaccinia Ankara, distinct from the licensed MVA-BN/JYNNEOS product) in NHPs further highlighted dosage selection biases that complicate cross-platform comparisons [83]. Human immunogenicity data for these candidates remain sparse, and extrapolation of animal efficacy to clinical effectiveness should be interpreted with caution. Modeling studies suggest that antibody decay kinetics in humans follow a two-phase pattern (fast decay t½ ≈ 21 days; slow decay t½ ≈ 1721 days), which may not be captured by short-term animal studies [84].

A primary obstacle in modern mpox vaccinology is the near-total reliance on inferred efficacy data, which presents a critical translational gap for clade I lineages. While real-world vaccine effectiveness estimates for MVA-BN (JYNNEOS) during the 2022 global outbreak were established between 36% and 86%, these metrics, as well as subsequent immunogenicity benchmarks, derive entirely from clade IIb exposure networks or historical vaccinia virus surrogates. Consequently, there is an absolute scarcity of direct human clinical efficacy data demonstrating protection against clade I strains. This data deficit has become an urgent public health concern following the emergence and sustained human-to-human transmission of clade Ib in the Democratic Republic of the Congo and neighboring regions, where case fatality rates have exceeded 10% in specific vulnerable settings [79].

To circumvent this clinical data vacuum, public health frameworks currently rely heavily on mathematical modeling and in vitro cross-neutralization assays. For instance, predictive models from non-endemic settings estimate that targeted reactive vaccination strategies could achieve up to 82% effectiveness (95% CI: 74–88%) against clade Ib, showing negligible variance between single- and two-dose regimens under localized household transmission dynamics [85]. However, because these simulations lack empirical validation from direct clade I clinical trials, they introduce profound epidemiological uncertainties. While in vitro pseudovirus assays suggest that antibodies elicited by clade IIb or traditional vaccinia antigens retain cross-neutralizing capabilities against clade I targets [79], actual clinical protection remains unconfirmed. Furthermore, as highlighted by the European Centre for Disease Prevention and Control (ECDC), assessing real-world vaccine performance and clinical severity profiles for clade Ib remains heavily confounded by restricted sample sizes and pervasive case-ascertainment biases in affected territories. To bridge these operational blind spots, future clinical trial protocols must pivot away from historical surrogates, prioritize geographically diverse patient enrollment within endemic regions, and mandate strain-specific genomic tracking as a primary study endpoint.

The 2022 emergency highlighted critical global vulnerabilities in vaccine supply chains, as existing manufacturing capacities for MVA-BN proved insufficient to meet demand, leading to dose-rationing and off-label intradermal administration [80]. While next-generation platforms like mRNA offer rapid scalability and adaptability, their high dependency on ultra-cold chain logistics presents a significant barrier to equitable distribution in resource-limited endemic regions. Furthermore, the production of complex multi-antigen vaccines essential for broad protection increases manufacturing costs and complicates quality control measures compared to monovalent candidates. Consequently, major regulatory and translational challenges remain; many promising mRNA, DNA, nanoparticle, and recombinant protein platforms are currently supported primarily by preclinical immunogenicity and challenge data, with limited human efficacy evidence available. Overcoming these implementation barriers to ensure equitable access in endemic settings will require the establishment of validated correlates of protection, standardized immunological endpoints, and robust clinical evaluation across diverse populations. Additionally, future studies should prioritize evaluating vaccine responses across diverse populations, including immunocompromised individuals, people living with HIV, children, and older adults, as differences in immune competence may influence vaccine-induced protection, durability, and optimal vaccination strategies. Strengthening these regulatory pathways while facilitating global collaboration and post-licensure surveillance will be essential to accelerate the deployment of safe, effective, and accessible precision mpox vaccines.

To overcome these challenges, future research must prioritize the identification of validated CoP, moving beyond simple nAb titers to include functional B-cell and T-cell memory signatures. Establishing international reference standards for serological assays is vital to enable cross-study comparisons of vaccine performance. Furthermore, the development of thermostable formulations and mucosal delivery systems (e.g., intranasal or intrarectal) could significantly enhance global vaccine equity and block transmission at primary sites of infection. Finally, the design of universal OPXV vaccines targeting highly conserved epitopes remains a critical long-term goal to provide preparedness for both known MPXV clades and unforeseen zoonotic emergencies.

## 11. Conclusions

The re-emergence of mpox as a global public health concern highlights the limitations of traditional vaccinia-based vaccination strategies and underscores the need for more targeted and adaptive vaccine development. Advances in antigen discovery, structural vaccinology, immunoinformatics, artificial intelligence, and systems immunology are now enabling a transition toward precision vaccinology, in which protective antigens, immune targets, and vaccine platforms can be rationally designed and optimized according to specific immunological requirements. Rather than relying solely on traditional whole-virus approaches, next-generation mpox vaccines have the potential to incorporate structurally defined antigens, engineered immunogens, and tailored delivery platforms to achieve enhanced safety, durability, and population-specific protection. Despite considerable advances in precision mpox vaccinology, several critical challenges remain. A major limitation is the absence of validated human immunological correlates of protection [15], which hinders the evaluation and direct comparison of emerging vaccine candidates. Future research should therefore prioritize the establishment of standardized immunological assessment frameworks, including validated neutralization assays, cellular immune endpoints, and longitudinal clinical studies integrating humoral, cellular, and systems immunology biomarkers. Such standardized frameworks will facilitate regulatory evaluation and accelerate clinical translation of precision-designed vaccines. Moreover, successful implementation of precision mpox vaccinology will require a comprehensive understanding of the complex relationship between viral evolution, antigenic diversity, host immunity, and immune correlates of protection. Continued genomic surveillance, integration of computational prediction with experimental validation, and refinement of immune profiling approaches will be essential to identify robust correlates that can guide vaccine optimization and accelerate regulatory evaluation. In this context, multidisciplinary integration between structural biology, computational modelling, vaccinology, immunology, and clinical research will be critical for translating rational vaccine designs into effective interventions. By synthesizing current knowledge on mpox structural immunology, nAb targets, and emerging vaccine platforms, this review provides a consolidated reference that may assist researchers in guiding rational vaccine design, identifying key antigenic targets, and advancing next-generation mpox vaccine development for improved outbreak preparedness.

## Figures and Tables

**Table 1:** Key structural proteins of monkeypox virus (MPXV) as candidate antigens for multivalent vaccine strategies and their role in viral pathogenesis [20].

MPXV Gene	MPXV Protein	VACV Ortholog	Virion Form	Structural Location	Biological Function	Role in Viral Pathogenesis	Evidence for Protective Immunity
M1R	M1	L1	MV	Membrane surface protein	Myristoylated protein involved in membrane fusion	Facilitates fusion between viral membrane and host membrane, enabling core release	Strong; included in mRNA, DNA, protein subunit and nanoparticle vaccines
A29L	A29	A27	MV	Surface attachment protein	Binds host cell glycosaminoglycans	Mediates attachment to heparan sulfate on host cells	Strong; major neutralizing target
E8L	E8	D8	MV	Surface glycoprotein	Host receptor binding protein	Interacts with chondroitin sulfate to facilitate attachment	Emerging protective target; nanoparticle vaccines
H3L	H3	H3	MV	Envelope surface protein	Viral attachment and membrane stability	Enhances virion binding to host cells	Strong antibody target: included in BNT166
A30L	A30	A30	MV	Membrane-associated protein	Structural component of virion membrane	Supports viral entry machinery	Candidate protective agent
A35R	A35	A33	EV	Outer envelope protein	Promotes actin-based virion movement	Facilitates cell-to-cell viral dissemination	Strong EV target
B6R	B6	B5	EV	Envelope glycoprotein	Complement control protein	Enhances viral dissemination and immune evasion	reduce viral spread and disease severity in orthopoxvirus animal challenge models. Inclusion as a key EV antigen in multi-antigen vaccine formulations.
A21L	A21	A21	MV	Virion membrane	Fusion complex protein	Participates in membrane fusion during entry	Identified in antigen screening studies
A28L	A28	A28	MV	Virion membrane	Essential fusion protein	Coordinates viral membrane fusion with host membrane	Conserved neutralizing target
L1R	L1	L1	MV	Viral membrane	Structural fusion protein	Required for efficient entry into host cytoplasm	

**Table 2:** Mpox Transmission and Route-Specific Vaccine Efficacy.

Target/Outcome	Relevance to Mpox Vaccination	Key Findings	References
Transmission Dynamics	Determines whether vaccines can interrupt person-to-person spread.	Mpox is transmitted primarily through direct skin-to-skin contact, mucosal exposure (oral and rectal), and close contact within sexual networks.	[19]
Infection Prevention	Measures vaccine ability to prevent infection after exposure.	Current vaccines reduce susceptibility to infection but generally do not provide sterilizing immunity.	[43]
Disease Severity and Lesion Burden	Assesses protection against clinical disease.	Vaccination is associated with fewer lesions, reduced systemic symptoms, and faster lesion resolution in breakthrough infections.	[8]
Viral Shedding and Onward Transmission	Evaluates the impact of vaccination on infectiousness.	Vaccinated individuals often exhibit lower lesion viral loads, although breakthrough shedding and onward transmission can still occur.	[9]
Mucosal Replication	Critical for preventing transmission at primary sites of infection.	Conventional subcutaneous vaccination induces limited mucosal IgA responses, allowing continued viral replication at oral and rectal mucosal surfaces.	[9]
Intradermal (ID) Vaccination	Alternative dose-sparing administration strategy.	Fractional intradermal dosing (0.1 mL) generates comparable systemic immunity to standard dosing while increasing local reactogenicity.	[44]
Intranasal (IN) Vaccination	Designed to enhance mucosal and respiratory immunity.	Preclinical studies suggest intranasal vaccination can induce mucosal immune responses and may reduce transmission more effectively than parenteral routes.	[9]

**Table 3:** Candidate immunological correlates of protection, biomarkers, and clinical endpoints relevant to precision mpox vaccinology.

Immune Domain	Candidate Biomarker	Measurement Method	Potential Clinical Relevance	Current Status	References
Neutralizing Antibodies	Neutralizing antibody titer	PRNT, pseudovirus neutralization assay	Prevention of infection and disease	Candidate CoP; not validated	[10]
Binding Antibodies	Anti-MV and anti-EV IgG	ELISA	Breadth of vaccine response	Candidate biomarker	[34]
Fc-Mediated Functions	ADCC activity	NK-cell activation assays	Clearance of infected cells	Experimental biomarker	[47,48]
	ADCP activity	Phagocytosis assays	Virion and infected-cell clearance	Experimental biomarker	
Complement Activity	ADCD and complement-mediated neutralization	Complement deposition assays	Enhanced viral neutralization	Experimental biomarker	[53]
Cellular Immunity	MPXV-specific CD4^+^ T cells	ELISpot, ICS, flow cytometry	Immune memory and antibody support	Candidate biomarker	[18]
	MPXV-specific CD8^+^ T cells	ELISpot, ICS, flow cytometry	Viral clearance and disease control	Candidate biomarker	[19]
Tissue Immunity	Tissue-resident memory T cells (TRM)	Tissue sampling, advanced flow cytometry	Rapid local protection	Emerging biomarker	[69]
Mucosal Immunity	Secretory IgA	Mucosal ELISA	Prevention of mucosal infection and transmission	Emerging biomarker	[39]
Viral Control	Viral load and shedding duration	qPCR, viral culture	Potential surrogate of transmission reduction	Candidate clinical endpoint	[5]
Clinical Protection	Lesion burden and healing time	Clinical assessment	Disease attenuation	Candidate efficacy endpoint	[14]
Human Correlate of Protection	None established	Not applicable	Not applicable	Major knowledge gap	[1,2]

**Table 4:** MVA-BN Vaccine Performance and Efficacy Synthesis.

Metric/Population	Impact, Efficacy, and Clinical Data	References
1-Dose vs. 2-Dose	Single doses confer 60–80% early protection; complete two-dose schedules are vital to solidify efficacy up to 85–90%+.	[72]
Intradermal vs. Subcutaneous	Clinical efficacy profiles remain virtually identical, though local skin induration and erythema are markedly elevated with ID.	[44]
Prior Smallpox Vaccination	Historical childhood vaccines leave long-lasting memory cells; modern MVA-BN acts as a strong anamnestic immune booster.	[16,31]
Durability (>12 months)	Circulating neutralizing titers decline steeply by 6–12 months, signaling the potential necessity for ongoing booster regimens.	[73]
Immunocompromised Populations	Generally safe in PLWH; optimal antibody responses require completion of the two-dose regimen.	[46]
Breakthrough Infections	Documented globally; typically associated with fewer lesions and milder clinical manifestations.	[74,75]

**Table 5:** Comparative Summary of Mpox Vaccine Candidates and Their Preclinical and Clinical Evidence Profiles.

Platform	Vaccine/Candidate	Developer	Antigen(s)	Model Tested	Endpoint Measured	Clinical Stage	Evidence Tier	Advantage	Major Limitations	References
Live Attenuated	ACAM2000	Emergent BioSolutions	Whole replicating VACV	Humans, NHPs	Neutralizing antibodies, cellular immunity, protection	Licensed	Clinical	Strong and durable humoral and cellular immunity; broad antigen repertoire including MV and EV antigens	Risk of adverse events, contraindicated in immunocompromised individuals, higher reactogenicity	[8]
Non-Replicating Viral Vector)	MVA-BN (JYNNEOS)	Bavarian Nordic	Whole MVA virus expressing orthopox antigens	Humans, NHPs	Neutralization, protection, T-cell responses	Licensed	Clinical	Strong safety profile, established regulatory pathway, broad orthopoxvirus protection	More expensive than conventional vaccines, requires cold storage	[44,70]
Live Attenuated (Minimally Replicating)	LC16m8	KM Biologics	Attenuated VACV	Humans, NHPs	Antibody responses, safety	Licensed in Japan	Clinical	Improved safety relative to ACAM2000 while maintaining strong immunity	Limited global availability and clinical experience	[71]
mRNA-LNP	BNT166	BioNTech	M1, H3, A35, B6	Preclinical/early clinical	nAb titers, immunogenicity	Phase 1/2	Moderate–High (early clinical immunogenicity)		Durability and correlates of protection not yet established	[7]
	mRNA-1769	Moderna	A29L, M1R, A35R, B6R	Preclinical/early clinical	nAb titers (>10^3^–10^4^ range), T-cell response	Phase 1/2	Moderate–High		Limited human efficacy data	[79]
mRNA multivalent	Multivalent MPXV mRNA vaccines	Academic/Industry groups	A29L, M1R, A35R, B6R	Mice, NHPs	Binding antibodies, neutralization, challenge protection	Preclinical	Animal challenge	Rapid redesign, scalable manufacturing, precise antigen selection, balanced MV/EV targeting	Cold-chain requirements, limited long-term human data	[20]
DNA vaccine	Quadrivalent DNA vaccines	Multiple academic groups	A27, L1, A33, B5 homologs	Mice, NHPs	Neutralization, survival after challenge	Preclinical	Animal challenge	Thermostable, low-cost production, easy storage and transport	Lower immunogenicity often requires electroporation or boosting	[13]
Recombinant Protein	Quadrivalent protein vaccines	Academic groups	A29L, M1R, A35R, B6R	Mice, NHPs	Antibody responses, challenge protection	Preclinical	Animal challenge	Excellent safety profile, straightforward regulatory pathway	Requires adjuvants and multiple doses, weaker T-cell immunity	[28]
Nanoparticle vaccine	Ferritin/self-assembling nanoparticle vaccines	Academic groups	MV and EV antigens displayed on nanoparticles	Mice	Neutralization, germinal center responses	Preclinical	Immunogenicity studies	Enhanced antigen presentation, potential dose sparing, balanced MV/EV immunity	Manufacturing complexity, limited clinical experience	[3]
Virus-Like Particle (VLP)	Experimental MPXV VLP vaccines	Academic groups	Multiple MPXV structural proteins	Mice	Antibody and T-cell responses	Preclinical	Immunogenicity studies	Mimics native virion structure without replication	Manufacturing challenges and limited efficacy data	[76]

**Table 6:** Comparative Assessment of Mpox Vaccine Platforms Based on Safety, Immunogenicity, and Deployment Feasibility.

Platform	Safety	Immunogenicity	Speed of Redesign	Manufacturing Scalability	Cold Chain	Suitability for Endemic Regions	Cost	Reactogenicity	Regulatory Feasibility	Balanced MV/EV Immunity
Live Attenuated	Moderate	Very High	Slow	Established but requires biological production systems	Moderate	Limited by safety concerns and monitoring requirements	Moderate	High	High	Excellent
Viral Vector (MVA)	High	High	Moderate	Scalable cell culture production	Moderate	Manufacturing complexity	Moderate–High	Moderate	High	Good–Excellent
mRNA	High	High	Very Fast	High	Often required frozen storage	Currently limited by cold chain infrastructure	Moderate–High	Moderate	Increasing	Excellent
DNA	High	Moderate	Fast	High	Minimal	Favorable for resource limited settings	Low	Low	Moderate	Good
Recombinant Protein	Very High	Moderate	Moderate	High	Moderate	Suitable due to favourable safety profile	Moderate	Low	High	Good
Nanoparticle	High	High	Moderate-fast	Moderate	Variable	Promising, though large-scale deployment remains limited	Moderate–High	Low–Moderate	Emerging	Excellent

## Data Availability

There is no data associated with this work. All data relevant to this review is included in the text and references.

## Abbreviations

The following abbreviations are used in this manuscript:

nAb	Neutralizing antibody
MPXV	Monkeypox virus
VACV	Vaccinia Virus
MV	Mature virion
EV	Enveloped virion
CoP	Correlates of protection
dsDNA	Double-stranded DNA
kb	Kilobases
IMV	Intracellular mature virion
EEV	Extracellular enveloped virion
EFC	Entry-fusion complex
NK	Natural Killer
IFNs	Interferons
PAMPs	Pathogen-associated molecular patterns
TLRs	Toll-like receptors
MAPK	Mitogen-Activated Protein Kinase
PKR	Protein kinase R
eIF2α	Eukaryotic translation initiation factor 2α
IRF	Interferon regulatory factor
OMCP	Orthopoxvirus Major Histocompatibility Complex (MHC) class I-like protein
CTLs	Cytotoxic T lymphocytes
NHP	Non-human primate
ADCC	Antibody-dependent cellular cytotoxity
ADCP	Antibody-dependent cellular phagocytosis
ADNP	Antibody-dependent neutrophil phagocytosis
ADCD	Antibody-dependent complement deposition
PLHIV	People living with HIV
hBD3	human β-defensin 3
PADRE	pan-HLA DR-binding epitope
DCs	Dendritic cells
LuS	Lumazine synthase
SNPs	Single nucleotide polymorphisms
FWPV	Fowlpox virus
